# Predictors of outcome in refractory generalized convulsive status epilepticus

**DOI:** 10.1002/epi4.12394

**Published:** 2020-04-22

**Authors:** Dilli Ram Kafle, Appu Jha Avinash, Aashish Shrestha

**Affiliations:** ^1^ Neurology Nobel Medical College Biratnagar Nepal

**Keywords:** disability, EEG, GCS, predictors, refractory status epilepticus

## Abstract

**Objective:**

Refractory status epilepticus is a serious condition in which seizure continues despite use of two antiepileptic medications. Retrospective studies have shown that 29%‐43% of SE patients progress into RSE despite treatment. Mortality following RSE is high. We aimed to evaluate the predictors of outcome in patients with RSE at a tertiary care center.

**Methods:**

Sixty‐eight consecutive patients with RSE who presented to our hospital between February 2018 and January 2020 were evaluated for outcome.

**Result:**

In our study 28(41.2%), patients who failed to respond to first‐ and second‐line antiepileptic drug responded to the third‐line antiepileptic drug thus avoiding mechanical ventilation and intravenous anesthesia. Low GCS at admission (*P* < .001), need for mechanical ventilation and intravenous anesthesia (*P* = .018), and long duration of RSE before recovery (*P* = .035) were strongly associated with worse outcome. Duration of RSE before starting treatment (*P* = .147), previous history of seizure (*P* = .717), and age of the patient (*P* = .319) did not influence the outcome.

**Significance:**

In our study, we prospectively evaluated patients with RSE and followed them for one month after discharge from the hospital. Unlike some of the previous studies, we identified an interesting finding whereby a significant proportion of the patients responded to the third‐line antiepileptic drug and thus avoiding the complications related to mechanical ventilation.

**Key Points**

- Long duration of RSE before starting treatment did not influence the outcome. However, long duration of RSE before recovery was associated with worse outcome.
- In patients with higher GCS and low STESS score, less aggressive approach with antiepileptic medication may be tried before proceeding to mechanical ventilation and induction of coma.
- A significant proportion of patients have improvement in their disability status even after discharge from the hospital.·

## 1 INTRODUCTION

Status epilepticus is an important neurological emergency characterized by significant morbidity and mortality requiring urgent management. The incidence of status epilepticus among adults is approximately 17.1 per 100 000 people per year.^1^ Generalized convulsive status epilepticus in adults has been defined as continuous seizure occurring for more than 5 minutes or two or more discrete seizures with incomplete recovery of consciousness between the seizures**.** ^2^

Refractory status epilepticus (RSE) is defined as seizure which continues to occur even after one first‐line medication (intravenous benzodiazepine) and one second‐line medication (intravenous phenytoin or valproic acid or phenobarbitone) have been given.^3^ Status epilepticus lasting more than 60 minutes has also been regarded as RSE. RSE has a worse prognosis than SE which responds rapidly to medication.^3^, ^4^ Retrospective studies have shown that 29%‐43% of SE patients progress into RSE despite treatment.^3^, ^5^, ^6^, ^7^ Risk factors for RSE include new‐onset seizures, focal motor seizures, and acute CNS disorders, such as encephalitis.^8^ New‐onset refractory status epilepticus (NORSE) is defined as new‐onset RSE in whom no obvious cause is identified in otherwise healthy individuals.^9^, ^10^

Etiology of SE and RSE includes CNS infections like encephalitis and neurocysticercosis.^11^ Other commonly noted etiologies include unknown, immunological, and cerebrovascular.

Single series and a meta‐analysis of patients with RSE have shown an association of mortality with older age, etiology, and seizure duration.^12^, ^13^ A meta‐analysis of 193 patients with RSE between 1980 and 2001 identified a mortality rate of 48%.^12^

For the treatment of SE, benzodiazepine especially IV lorazepam or IM midazolam is followed by second‐line therapies which include fosphenytoin, valproic acid, and levetiracetam. There is no clear evidence that any one of these options is better than the other.^14^ The second‐line drugs appear to be equally effective.^15^ As there is lack of a clear evidence‐based guideline for managing RSE, they are managed as per the clinical situation with early induction of pharmacological coma with midazolam, propofol, or barbiturates.^16^

Most of our knowledge on RSE depends on retrospective studies, while its management is based on small series and expert opinions. We performed this study to evaluate the frequency, risk factors, and outcome in patients with RSE.

## 2 MATERIALS AND METHODS

We conducted an observational prospective study in all convulsive RSE patients presenting to the emergency room or those patients who were admitted to the ICU or neuro ward for medical or surgical problem and developed refractory status epilepticus. This study was conducted between February 2018 and January 2020 at Nobel Medical College Biratnagar, which is a tertiary care hospital in the eastern part of Nepal. The resident posted in neurology department, and the investigator was actively involved in the management and follow‐up of the patients. A systematic protocol was followed for the management of status epilepticus. Intravenous lorazepam or IM midazolam was the first‐line drug used. Intravenous phenytoin or levetiracetam was the most often used second‐line agent. In those who failed to respond to second‐line drug, we used another second‐line drug (either phenytoin or levetiracetam) instead of going straight to mechanical ventilation and induction of coma. So in RSE in whom phenytoin was given as the second‐line drug, levetiracetam was chosen as third‐line drug and vice versa. We chose phenytoin because it is a time‐tested drug in controlling status epilepticus. Levetiracetam was chosen because of better safety profile in terms of side effects and has been reported to be equally efficacious as valproic acid.^15^ Valproic acid was used as a fourth‐line agent in patients in whom mechanical ventilation could not be done because of comorbid condition or when the family member was reluctant to undergo aggressive management with induction of coma. Status Epilepticus Severity Scale score was used to access the severity of SE. EEG was done in all the patients and repeated frequently when the seizure was ongoing.

Inclusion criteria: Patients who were 16 years and older, in a state of refractory status epilepticus and given consent for the study. Status epilepticus was defined as the seizure occurring continuously for more than 5 minutes or recurrent seizure without regain of consciousness in between the attacks. RSE was defined when the seizure failed to respond to one first‐line and a second‐line drug and also when the seizure continued for more than 1 hour.

The modified Rankin scale (MRS) was obtained at discharge and 1 month after discharge. Poor functional outcome was defined as MRS score of four or higher at discharge. Outcome of the patient was assessed during follow‐up visit of the patient or through telephone.

In high‐risk patients who did not respond to third‐line AED, endotracheal intubation and IV anesthesia were started. Intravenous midazolam infusion and thiopental were the agents used for induction of coma. EEG was monitored to achieve burst suppression.

Neuroimaging (either CT scan or MRI) of the brain was done in all the patients to look for any structural abnormality as the cause of seizure. Lumbar puncture was done when the neuroinfection was the likely cause of epilepsy. Complete blood count, electrolyte, and renal and liver function test were done in all the patients. Etiology of SE was classified according to the ILAE criteria as acute symptomatic, remote symptomatic, those with preexisting epilepsy, and idiopathic.^17^ Acute symptomatic group included patients with acute cerebrovascular insult (ischemic/hemorrhagic stroke), CNS infections (encephalitis and neurocysticercosis), and those with metabolic derangement. The information collected included demographic profile, previous history of epilepsy, family history of epilepsy, neuroimaging finding, duration of SE before arrival to hospital, AED used, response to medication, need for mechanical ventilation, outcome of the patient at discharge, and after one‐month follow up.

Data were entered in SPSS version 16 for processing and analysis of our data. All data were analyzed for normality. Categorical data were evaluated for significance with chi‐square test or Mann‐Whitney *U* test. Student's *t* test and Pearson correlation test were used to analyze quantitative data. *P* Value < .05 was considered statistically significant.

## 3 RESULTS

Mean age of the study population with RSE who were admitted to our hospital was 45.13 (Table 1). However, mean age of onset of the first seizure was 41.06 years. While previous history of status epilepticus was given by 23.5%, most of our patients who presented in the state of refractory convulsive RSE never had a history of seizure (74.5%). Only 35.3% of the patients previously had seizure. GCS and STESS score were calculated at presentation to the hospital. Though we gave a third‐line AED before proceeding to mechanical ventilation and beginning of IV anesthesia, 44.1% of the patients had to ultimately be intubated because the seizure continued. However, we were able to achieve seizure control in additional 28 (41.2%) patients with third‐line AED thus avoiding mechanical ventilation and general anesthesia. Mean duration for recovery of the RSE was 3.2 days. Neuroimaging was abnormal in 41% of patients which showed finding suggestive of encephalitis or cerebral infarction as more common cause. Mean duration of the hospital stay was 11.4 days. There were total 12 (17.65%) deaths. Ten patients who underwent mechanical ventilation died, while two patients who were not intubated due to comorbid condition but treated only with AED died. Most of our patients had gradual recovery in their MRS after discharge from hospital when we followed them for 1 month.

**TABLE 1:** Characteristics of patient with RSE

Characteristics	Categories	No. of patient	Percentage
Age‐group in years	<60	49	72.1
	≥60	19	27.9
Mean age in years ± SD (Min‐Max)		45.13 ± 20.10 (18‐84)	
Gender	Male	40	58.8
	Female	28	41.2
Mean age in years at seizure onset		41.06 ± 22.50 (2‐84)	
Median duration in days of SE before treatment		1 (1‐2) (0.01‐7.0)	
Median stress score (IQR) (Min‐Max)		3 (2‐4) (1‐5)	
Median GCS (IQR) (Min‐Max)		10 (5‐13) (3‐15)	
Precipitating factor	Present	4	5.9
	Absent	64	94.1
Number of drugs used to treat SE	2	2	2.9
	3	46	67.6
	4	19	27.9
	5	1	1.5
Median drugs used to treatment SE (IQR) (Min‐Max)		3 (3‐4) (2‐5)	
MV and IV anesthesia	Yes	30	44.1
	No	38	55.9
Complications	Normal	39	57.4
	DVT	3	4.4
	UTI	13	19.1
	Bedsore	7	10.3
	Pneumonia	6	8.8
CT/MRI	Normal	40	58.8
	Abnormal	28	41.2
Median duration of SE before recovery (IQR) (Min‐Max)		2 (1‐5) (0.015‐18.0)	
Outcome	Alive	56	82.4
	Death	12	17.6
Total		68	100.0

Twenty‐nine patients developed some complications during the hospital stay. Three patients developed deep venous thrombosis. Thirteen patients developed urinary tract infection. Seven patients developed, and six patients developed pneumonia (Table 2).

**TABLE 2:** Etiology of RSE

Variable	Number of patients
Encephalitis	19 (28%)
Cerebral infarct	13 (19%)
Idiopathic(unknown cause)	11 (16%)
Metabolic	5 (7.4%)
Neurocysticercosis	4 (5.9%)
Cerebral atrophy	3 (4.4%)
SLE	2 (3%)
Cerebral hypoxia	2 (3%)
Gliosis	2 (3%)
Mesial temporal sclerosis	2 (3%)
ADEM	1 (1.5%)
PRES	1 (1.5%)
Tubercular meningitis	1 (1.5%)
Subdural hematoma	1 (1.5%)
Hydrocephalus	1 (1.5%)

Encephalitis was found to be the most common cause of RSE accounting for 28% of cases followed by cerebral infarct. When no cause for RSE was identified, it was labeled was idiopathic which was found in 16%. Though we did workup for viral etiology of encephalitis, treatment of autoimmune encephalitis was largely based upon clinical judgment as most of our patients could not afford for the test. Neurocysticercosis has been found to be the most common identified cause of seizure in Nepal. However, it accounted for only 5.9% of RSE.

Mean MRS score at discharge was 2.9. There was improvement in the MRS over the following 1‐month period with mean MRS at 1 month of 2.4 which was statically significant (*P* < .001).

Low GCS at admission was strongly associated with worse clinical outcome as shown in Table 3. Duration of SE before treatment, age at seizure onset, number of drugs needed to treat SE, and length of hospital stay did not have any impact on the outcome. STESS score and duration of RSE before recovery influenced the outcome as shown in Tables 4 and 5.

**TABLE 3:** Predictors of outcome

Variables	Alive		Death		*P* value	Remarks
	Mean	SD	Mean	SD		
Age in years	44.00	19.32	50.42	23.64	.319	NS
GCS At admission	10.07	3.99	5.50	2.61	<.001	Sig

**TABLE 4:** Outcome in patients with RSE

Variables	Alive			Death			*P* value	Remarks
	Median	Q1	Q3	Median	Q1	Q3		
Duration of SE before treatment	1.00	1.00	2.00	1.00	0.63	1.00	.147	NS
Age at seizure onset	38.00	19.75	54.75	54.50	21.50	73.50	.330	NS
STESS Score	2.00	2.00	3.00	3.50	3.00	5.00	.002	Sig
Number of drugs used to treat SE	3.00	3.00	4.00	3.00	3.00	4.00	.969	NS
Duration of SE before recovery	1.50	1.00	3.75	3.00	2.00	5.75	.035	Sig
Length of hospital stay	10.00	6.25	14.75	9.00	3.50	15.25	.311	NS

**TABLE 5:** Predictors of outcome in RSE

Characteristics	Categories	Outcome		*P* value	Remarks
		Alive	Death		
Age‐groups in years	<60	43	6	.080	NS
	≥60	13	6		
Gender	Male	32	8	.748	NS
	Female	24	4		
Past history of seizure	Present	21	3	.518	NS
	Absent	35	9		
Past history of SE	Present	14	2	.717	NS
	Absent	42	10		
Family history of seizure	Present	3	1	.549	NS
	Absent	53	11		
Precipitating factor	Present	2	2	.141	NS
	Absent	54	10		
MV and IV anesthesia	Given	21	9	.018	Sig.
	Not given	35	3		
DVT	Absent	54	11	.447	NS
	Present	2	1		
UTI	Absent	46	9	.687	NS
	Present	10	3		
Bedsore	Absent	51	10	.598	NS
	Present	5	2		
Pneumonia	Absent	51	11	.947	NS
	Present	5	1		

**FIGURE 1:**
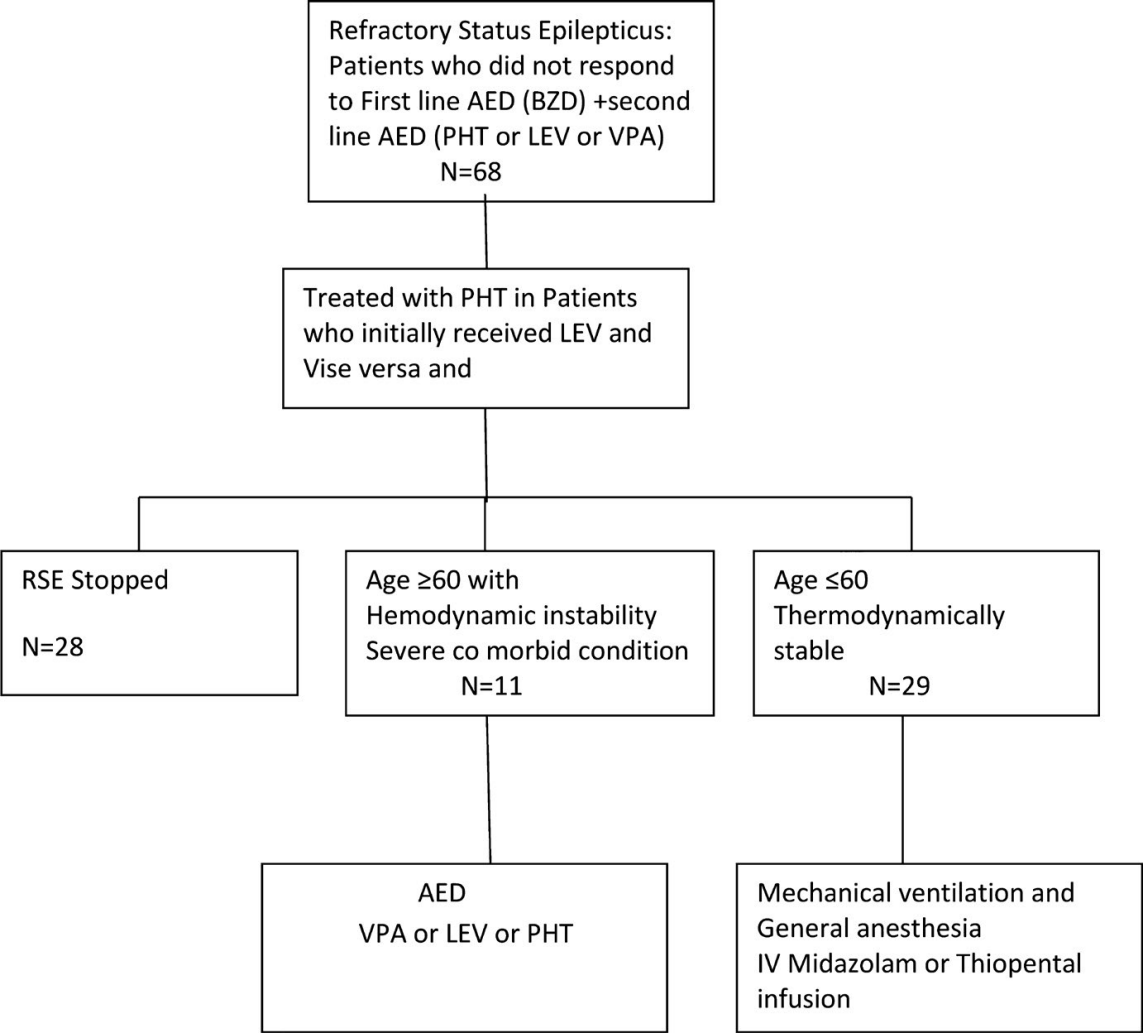
Sixty‐eight patients failed to respond to the first‐line drug (either intravenous lorazepam or midazolam) and a second‐line drug (intravenous phenytoin or levetiracetam or valproic acid) and were regarded as refractory status epilepticus (RSE). In those patients with RSE, we used either phenytoin or levetiracetam as the third‐line drug before proceeding to endotracheal intubation and induction of coma. Twenty‐eight patients responded to the third‐line antiepileptic. Eleven patients who did not respond to the third‐line antiepileptic but were unsuitable for general anesthesia were given fourth‐line drug. Twenty‐nine patients who were young and suitable for general anesthesia underwent mechanical ventilation and induction of coma using either midazolam or thiopental infusion. Two patients treated without anesthesia died. Ten patients managed with mechanical ventilation and general anesthesia died. AED, antiepileptic; LEV, levetiracetam; PHT, phenytoin; VPA, valproic acid

Age at onset of RSE, sex, previous history of epilepsy, previous history of SE, family history of SE, or the presence of complications did not influence the outcome. However, need for mechanical ventilation was significantly associated with worse outcome (Figure 1).

## 4 DISCUSSION

Our study included all the consecutive patients admitted with convulsive RSE over a period of 2 years. Patients with low GCS were managed in intensive care unit, while those with good GCS and low STESS score were managed in neuro ward. Infection of the CNS was the most important cause of RSE with encephalitis being the most common. This finding is similar to the previous studies.^3^, ^5^ Other infectious causes of RSE were tubercular meningitis and neurocysticercosis. Besides neuroinfection, cerebral infarct and unknown factors were important cause of RSE.

Most of our patients did not have history of epilepsy which did not influence the outcome (*P* = .518). History of SE was reported by minority of patients. This is similar to the findings of a study by Hernandez et al that found that having prior history did not influence the outcome in patient with RSE.^18^

Age at RSE did not have impact on outcome (*P* = .33). Study by Hocker SE et al also did not find any association between age and outcome.^19^

One important finding in our study was that 28 (41.2%) of our patients responded to a third‐line AED which in most cases was either phenytoin or levetiracetam. This finding is in contrast to that of the VA Cooperative Study in which only 5.3% of patients responded to phenobarbitone after failing to show any response to treatment with lorazepam and phenytoin.^20^ In our study, we included patients with only convulsive RSE, while the former study included both nonconvulsive and convulsive RSE. While in VA Cooperative Study phenobarbitone was used as the third‐line drug, we used levetiracetam as the third‐line drug in those patients who received phenytoin as the second‐line drug and vice versa. Recent studies have shown that levetiracetam is as good as phenytoin in the treatment of RSE.^15^

We did not find any association between duration of SE before starting treatment and outcome (*P* = .147). The mean duration of SE before arrival to our hospital and starting treatment was 1.4 days. As Nepal is a hilly country and people have limited access to tertiary care hospitals, most patients were brought late due to long distance they had to travel before coming to our hospital. Similar finding has been observed in some of the previous studies.^21^, ^22^, ^23^

Though time to starting treatment for SE did not have significant influence on outcome, duration of RSE before recovery was associated with worse outcome in our study (*P* = .035).

Need for mechanical ventilation and IV anesthesia was strongly associated with worse outcome (*P* < .001). However, duration of mechanical ventilation had no influence on MRS (*P* = .39). Outcome was measured as either dead or alive one month after discharge from the hospital, while MRS indicated degree of disability during follow‐up. Mechanical ventilation was used in patients who failed to respond to third‐line AED and thus represented more severe form of RSE with worse outcome than patients with RSE controlled with only AED. Intravenous midazolam was used first for induction of general anesthesia. In patients who failed to respond to midazolam, thiopental was used until seizure was controlled or significant side effect limited its use.

We calculated STESS score in all the patients presenting as RSE and evaluated their influence on outcome and MRS score 1 month after discharge. High STESS score was strongly associated with worse outcome in terms of mortality (*P* = .002) and MRS score. STESS is an excellent predictor of outcome: Patients with a low score have a reliably good prognosis for survival, as well as for return to baseline clinical condition.^24^ We also found low GCS at admission to be associated with worse clinical outcome (*P* < .001). Low GCS and coma were associated with higher mortality, worse clinical outcome, and longer duration of hospital stay in an earlier study.^18^

Number of drugs needed to treat SE did not influence outcome (*P* = .969). Development of complication during hospital stay affected both the outcome and prolonged the duration of hospital stay (*P* < .001). Urinary tract infection was the most common complication observed in our patients. Hypotension, deep venous thrombosis, and pneumonia were other complications observed in our patients which significantly contributed to worse outcome.

Modified Rankin Scale was used to access the functional outcome at discharge and also 1 month after discharge. Nine patients died during the hospital stay, while 3 patients died after being discharged during the follow‐up period of 1 month. We observed a total of 12 (17%) mortality in patients of RSE. Duration of hospital stay was not associated with worse outcome (*P* = .311).

## 5 STRENGTH AND LIMITATION

We prospectively studied a large number of patients with convulsive RSE. Contrary to the current trend in treatment, a large number of our RSE patients responded to the third‐line AED and became seizure‐free, thus avoiding mechanical ventilation. Our study has some limitation. As we collected data from single center, a selection bias may be present. Unlike other studies which excluded anoxic patients, we included all the patients with convulsive RSE. We did lumbar puncture and CSF analysis in all patients with suspected encephalitis. We did serological testing for viral etiology but could not do workup for autoimmune encephalitis as this test was not available in our center.Most of our patients could not afford to test for autoimmune panel. So treatment for autoimmune encephalitis was largely based on clinical judgment of the investigator. Patients who needed mechanical ventilation developed more complications like pneumonia, urinary tract infection, and bedsore with long‐term consequences.

## CONFLICT OF INTEREST

None of the authors involved in this study has any conflict of interest. We confirm that we have read the Journal's position on issues involved in ethical publication and affirm that this report is consistent with those guidelines.
